# Genetic mapping reveals the complex genetic architecture controlling slow canopy wilting in soybean

**DOI:** 10.1007/s00122-024-04609-w

**Published:** 2024-04-17

**Authors:** Ethan Menke, Clinton J. Steketee, Qijian Song, William T. Schapaugh, Thomas E. Carter, Benjamin Fallen, Zenglu Li

**Affiliations:** 1 Institute of Plant Breeding, Genetics, and Genomics, and Department of Crop and Soil Sciences, University of Georgia, Athens, GA USA; 2 Soybean Genomics and Improvement Laboratory, USDA-ARS, Beltsville, MD USA; 3 Department of Agronomy, Kansas State University, Manhattan, KS USA; 4 Department of Crop and Soil Sciences, North Carolina State University and USDA-ARS, Raleigh, NC USA

## Abstract

In soybean [*Glycine max* (L.) Merr.], drought stress is the leading cause of yield loss from abiotic stress in rain-fed US growing areas. Only 10% of the US soybean production is irrigated; therefore, plants must possess physiological mechanisms to tolerate drought stress. Slow canopy wilting is a physiological trait that is observed in a few exotic plant introductions (PIs) and may lead to yield improvement under drought stress. Canopy wilting of 130 recombinant inbred lines (RILs) derived from Hutcheson × PI 471938 grown under drought stress was visually evaluated and genotyped with the SoySNP6K BeadChip. Over four years, field evaluations of canopy wilting were conducted under rainfed conditions at three locations across the US (Georgia, Kansas, and North Carolina). Due to the variation in weather among locations and years, the phenotypic data were collected from seven environments. Substantial variation in canopy wilting was observed among the genotypes in the RIL population across environments. Three QTLs were identified for canopy wilting from the RIL population using composite interval mapping on chromosomes (Chrs) 2, 8, and 9 based on combined environmental analyses. These QTLs inherited the favorable alleles from PI 471938 and accounted for 11, 10, and 14% of phenotypic variation, respectively. A list of 106 candidate genes were narrowed down for these three QTLs based on the published information. The QTLs identified through this research can be used as targets for further investigation to understand the mechanisms of slow canopy wilting. These QTLs could be deployed to improve drought tolerance through a targeted selection of the genomic regions from PI 471938.

**Supplementary Information:**

The online version contains supplementary material available at 10.1007/s00122-024-04609-w.

## Introduction

Soybean [*Glycine max* (L.) Merr.] is the largest oilseed crop globally, providing over a quarter of the vegetable oil and almost ~ 70% of the plant protein meal used worldwide. Global demand for soybean has led it to be the second most cultivated row crop in the USA, with an estimated 33.6 million hectares of soybean planted in 2020 (SoyStats 2020). Even with its economic importance, less than 10% of soybean hectares in the USA is under irrigation (Specht et al. 2015). Lack of irrigation leaves soybean extremely vulnerable to drought stress, which can cause more than a 40% reduction in yield (Specht et al. 1999; Purcell and Specht 2004).

Canopy wilting is caused by a decrease in turgor pressure in the soybean leaves and is a trait commonly used by soybean breeders to identify differential responses to stress. Slow or delayed canopy wilting has been observed in exotic soybean germplasm and is controlled by multiple plant mechanisms. A maturity group (MG) VI plant introduction (PI) 416937 from Japan has been observed to have slower canopy wilting under drought conditions than other existing cultivars (Sloane et al. 1990). PI 416937 also has an extensive lateral root system, with a large root surface area (Hudak and Patterson 1996; Pantalone and Rebetzke 1996) combined with low stomatal conductance (Tanaka et al. 2010). Fletcher et al. (2007) showed that under high vapor pressure deficit (VPD), PI 416937 reached a maximum transpiration rate at 2.0 kPa, while commercial cultivars showed increased transpiration rates at VPD greater than 2.0 kPa. This decreased transpiration at high VPD allows for the conservation of moisture, thus increasing the water use efficiency of the plants (Fletcher et al. 2007). The above results indicate that PI 416937 uses water conservation as its mechanism of the slow wilting phenotype that may protect yield under drought conditions.

PI 471938, an accession from Nepal, is an MG V introduction that exhibits slow canopy wilting as well, but the mechanism for this response to drought stress is unknown (Sadok et al. 2012; Bagherzadi et al. 2017). PI 471938 has shown normal nitrogen fixation under soil drying conditions (Sinclair et al. 2000; Devi and Sinclair 2013; Riar et al. 2018). It has been used by multiple southern breeding programs to develop cultivars in the Southeastern US (Devi et al. 2014; Carter et al. 2016). Based on pedigree data of lines that appeared in the USDA Uniform Tests, cultivar registrations, and plant variety protection applications, PI 471938 is a parent of six varieties developed from the population (Hutcheson × PI 471938) used in this experiment and is in the ancestry of at least 25 other breeding lines that reached the Uniform Yield Tests (Soybase.org, 2021). In addition, PI 567690 and PI 567731, both MG III, have been identified as two new sources of slow canopy wilting for early maturity group soybeans (Pathan et al. 2014; Ye et al. 2020).

Several studies of canopy wilting have been performed to understand the underlying genetics using bi-parental populations and genome-wide association studies. Kaler et al. (2017) used 373 MG IV soybean genotypes as a genome-wide association panel to identify genomic regions associated with slow canopy wilting. In this study, the authors found 61 single nucleotide polymorphisms (SNPs) that tagged 51 loci on 19 of the 20 soybean chromosomes (Kaler et al. 2017). Steketee et al. (2020) used a panel of 162 MG VI-VIII accessions and cultivars to identify genomic regions associated with canopy wilting. The study identified 45 unique SNPs related to differential canopy wilting at 44 loci in this population (Steketee et al. 2020). Twenty genomic regions on chromosomes (Chrs) 1, 4, 6, 9, 12, 15, 18, and 19 from Steketee et al. (2020) were also identified by Kaler et al. (2017). Using GWAS, Chamarthi et al. (2021) confirmed 31 slow wilting loci identified previously by Kaler et al. (2017) and Steketee et al. (2020). Abdel-Haleem et al. (2012) used a recombinant inbred line (RIL) population derived from a cross of ‘Benning’ (a fast wilting MG VII cultivar) × PI 416937 to identify seven quantitative trait loci (QTLs). These QTLs explained 75% of the phenotypic variation observed in canopy wilting using multiple interval mapping (Abdel-Haleem et al. 2012). Of the seven QTLs identified in multiple locations, five QTLs on Chrs 2, 4, 5, 12, and 19 inherited the favorable alleles from PI 416937 for the slow canopy wilting trait. The two remaining QTLs identified in this population on Chrs 14 and 17 inherited the favorable alleles from the fast wilting cultivar Benning.

Charlson et al. (2009) investigated the effects of drought stress on a RIL population developed from a cross of ‘KS4895’, a fast wilting cultivar, and ‘Jackson’, a slow wilting cultivar. Four QTLs that explained 47% of the phenotypic variation in canopy wilting were identified on Chrs 8, 13, 14, and 17. The slow canopy wilting cultivar, Jackson, is present in the pedigree of the fast wilting cultivar Benning, which could explain the beneficial drought tolerance alleles identified from Benning in the Benning × PI 416937 RIL population (Charlson et al. 2009; Abdel-Haleem et al. 2012). Using five RIL populations involving three slow wilting genotypes Jackson, PI 424140, and PI 416937, Hwang et al. (2015) found seven QTL clusters on Chrs 2, 5, 8, 11, 17, and 19 based on 95% confidence intervals from at least two mapping populations (Hwang et al. 2015). The populations from the earlier study used by Hwang et al. (2015) were used to perform a meta-QTL analysis, which identified nine meta-QTLs in eight QTL clusters on Chrs 2, 5, 11, 17, and 19 with a reduced confidence interval (Hwang et al. 2016). Ye et al. (2020) mapped the QTLs in two RIL populations derived from PI 567690 and PI 567731, two MG III exotic landraces. In the ‘Pana’ × PI 567690 RIL population, eight QTLs were identified, which were located at similar chromosomal positions to the QTLs identified in both Abdel-Haleem et al. (2012) and Hwang et al. (2016). Two QTLs on Chrs 6 and 10 that were identified in the ‘Magellan’ × PI 567731 were not identified in previous QTL mapping studies. PI 471938 has been utilized in the breeding programs as a source of slow canopy wilting, contributing to the development of numerous soybean germplasm and cultivars. Although slow canopy wilting QTLs have been reported in several studies (Charlson et al. 2009; Abdel-Haleem et al. 2012; Hwang et al. 2015; Kaler et al. 2017), in this research, we tried to understand and determine the genetic architecture controlling the slow canopy wilting trait from PI 471938 to support ongoing drought tolerance breeding efforts. The objectives of this study were to i) evaluate a RIL population in repeated field experiments for canopy wilting and ii) elucidate genomic regions responsible for slow canopy wilting in PI 471938.

## Materials and methods

### Plant materials

A cross between ‘Hutcheson’ (PI 518664) and PI 471938 was made in 1998 in Raleigh, NC, USA. Hutcheson is an MG V cultivar developed by Virginia Tech (Buss et al. 1988). PI 471938 is an MG V plant introduction characterized previously as a slow wilting soybean accession (Carter et al. 1999; Hufstetler et al. 2007; Sadok et al. 2012). The F_1_ seed from this cross was grown at the USDA Tropical Agricultural Research Station in Isabela, Puerto Rico. The F_2_ to F_4_ generations were advanced by single seed descent (Brim 1966) throughout the inbreeding process. The F_4_ plants were harvested individually and used to develop the 130 F_4_-derived recombinant inbred lines (RILs) used in this study.

### Evaluation of canopy wilting

The Hutcheson × PI 471938 RIL population was evaluated in Athens, GA (2016_GA) and Salina, KS (2016_KS) in 2016. Two-row plots were planted at both locations with three replications in GA and two replications in KS using a randomized complete block design. In 2018, the RIL population was evaluated in Midville, GA (2018_GA), Salina, KS (2018_KS), and Sandhills, NC (2018_NC). In 2019, the population was evaluated in Midville, GA (2019_GA), Salina, KS (2019_KS), and Sandhills, NC (2019_NC). The 2018 and 2019 experiments were planted as two-row plots in a randomized complete block design, with three replications. All environments were planted with 0.76 m row spacing at a seeding density of 32 seed m^−2^. A summary of phenotyping locations for the Hutcheson × PI 471938 RIL population is listed in Supplementary Table S1.

Canopy wilting was rated in increments of five on a scale from 0 to 100: 0 = no wilting present; 20 = slight wilting and some rolling in the top of the canopy; 40 = somewhat severe leaf rolling at the top of the canopy, moderate wilting of leaves throughout the rest of the canopy, and some loss of petiole turgidity; 60 = severe wilting of leaves throughout the entire canopy, with advanced loss of petiole turgidity; 80 = plants with petioles severely wilted and dead leaves throughout much of the canopy; and 100 = plant death.

The RILs were evaluated for canopy wilting by taking the mean of three ratings as the phenotypic score for the 2016_GA environment. A single rating was used as the phenotypic score for the 2016_KS environment. In 2017, an evaluation of this RIL population at three locations (Athens, GA, Salina, KS, and Sandhills, NC) was attempted, but no canopy wilting scores were recorded because of minimal water stress. In 2018, no canopy wilting evaluations were performed in Sandhills, NC, due to a lack of drought stress during the growing season. One wilting rating was taken at the 2018_GA environment. One wilting rating was collected in the 2018_KS environment for QTL mapping. The 2019_GA environment was rated three times during the growing season, with one of three ratings being used for mapping. A single rating for 2019_KS and 2019_NC environments was collected in both locations (Supplementary Table S1).

### Genotype data and quality control

DNA was extracted from leaf tissue and genotyped with the SoySNP6K iSelect BeadChip (Song et al. 2020). The leaf tissue collection and DNA extraction procedures were the same as described in Steketee et al. (2020). These genotyping efforts generated 5403 genome-wide SNPs that were analyzed using GenomeStudio software (Illumina Inc., San Diego, CA, USA) to perform SNP quality control for segregation distortion and compression of genotype calls. Monomorphic markers between the two parents were removed, leaving 1258 polymorphic SNP markers available to create a genetic map. Forty-six additional markers that did not meet requirements for joining a linkage group during the genetic map construction were removed, leaving a total of 1212 polymorphic SNP markers to be used for the genetic map construction for QTL mapping.

### Statistical analyses

Analysis of variance (ANOVA) was conducted using PROC MIXED in SAS version 9.4 (SAS Institute 2014). The model for the combined analysis was built by treating genotype, environment, genotype by environment interaction, and replication within the environment as random variables using the Standard Least Squares personality and REML method. Genotype means were separated by Fisher's least significant difference (LSD) test at the α = 0.05 probability level. Broad-sense heritability was calculated on an entry-mean basis according to Holland et al. (2003), with the variance components being calculated using a model where all variables were treated as random. Correlations of genotype means were calculated using PROC CORR in SAS version 9.4. Best linear unbiased predictions (BLUPs) were calculated for canopy wilting scores across all environments using SAS version 9.4. For individual environments only, genotype and replication were used and treated as random variables to calculate BLUPs. Using BLUP values for each genotype across and within environments helped to account for variation caused by environmental factors and missing data. BLUPs were used as the phenotypic values for subsequent QTL analyses.

### Genetic map construction and QTL analysis

The 1212 polymorphic SNP markers for the Hutcheson × PI 471938 RIL population were used to construct a genetic map in JoinMap 4.1 (Van Ooijen 2006). The logarithm of odds (LOD) criterion of greater than six was used to establish linkage groups. As necessary, some groups were then forced together to form 20 linkage groups based on the known chromosomes and physical positions of the SNP markers. Maximum likelihood (ML) mapping with the default settings was used to convert recombination frequencies into map distances in centiMorgans (cM). These cM positions were then used in subsequent QTL mapping.

The software package Windows QTL Cartographer (WinQTLCart) 2.5 (Wang et al. 2012) was used for composite interval mapping (CIM) using Model 6 of the Zmapqtl program module. The genome was scanned with a walking speed of 0.5 cM and a window size of 10 cM, and the forward–backward regression method was used to choose cofactors. The significance LOD threshold was determined by 1000 permutations, with a significance level of α = 0.05. The significance threshold LOD = 3.3 was used for the combined analysis. Significant QTLs were identified by the peak of the QTL meeting or exceeding the LOD score. The position of QTL peaks was determined by the highest score on a specific chromosome. MapChart 2.32 (Voorrips 2002) was used to visualize the genetic maps and QTL mapping results.

The peak SNPs of the QTLs on Chrs 2, 8, and 9 identified through composite interval mapping were used to identify nearby candidate genes. Song et al. (2020) defined genome-wide haplotype blocks for cultivated soybean based on a data set of 14,183 *G. max* accessions genotyped with the SoySNP50K assay. The confidence interval based on Glyma.Wm82.a2 physical position of each QTL was used to search the candidate genes associated with slow canopy wilting QTLs. A list of informative candidate genes and their annotations within the confidence interval for each QTL were examined based on the gene functional annotation deposited on Phytozome (https://data.jgi.doe.gov/refine-download). Candidate gene analysis was based on known genes related to drought resistance as reported by others.

## Results

### Genetic variation of canopy wilting for the RIL population

**Fig. 1:**
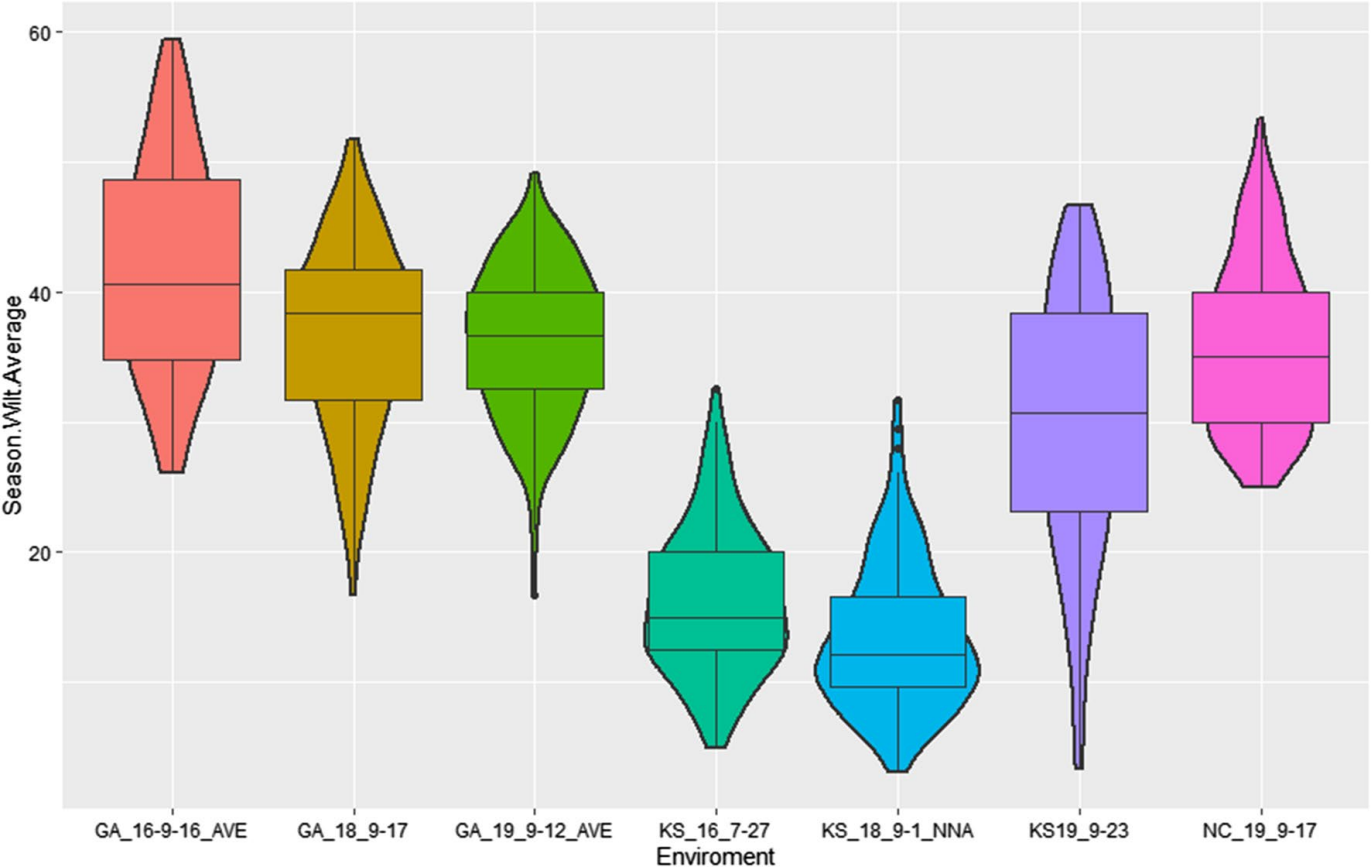
Distribution of canopy wilting scores for the recombinant inbred line population across the environments. Environments are named as Season.Wilt.Average, with Georgia (GA), Kansas (KS), and North Carolina (NC) as locations

The RIL population exhibited a wide range of canopy wilting among the RILs, and the wilting was more severe in the Georgia environments (Fig. 1). Genotypes, environments, and their interactions were statistically significant (*p* < 0.05) for canopy wilting scores (Supplementary Table S2). Correlation between the environments canopy wilting scores ranged from *r* =  − 0.17 to 0.44 (Table 1). The broad-sense heritability of canopy wilting on an entry mean basis for the combined environments was 0.29. No RILs in the combined environments had a lower mean wilting score than the slow wilting parent, PI 471938. Twenty-three of the RILs had higher canopy wilting based on mean performance in the combined environments as compared to the fast wilting parent, Hutcheson.

**Table 1:** Pearson correlations of canopy wilting scores among the environments

	2016_KS	2018_GA	2018_KS	2019_GA	2019_KS	2019_NC
2016_GA	0.07	0.20*	0.15	0.21*	0.25**	0.26**
2016_KS		− 0.08	0.15	0.19*	− 0.09	− 0.05
2018_GA			0.22*	− 0.17	0.22*	0.13
2018_KS				0.44***	0.38***	0.25**
2019_GA					0.25**	0.29***
2019_KS						0.38***

***﻿, **, and * indicate significance at < 0.0001, 0.01, and 0.05, respectively

### QTL mapping of canopy wilting trait for the RIL population

**Fig. 2:**
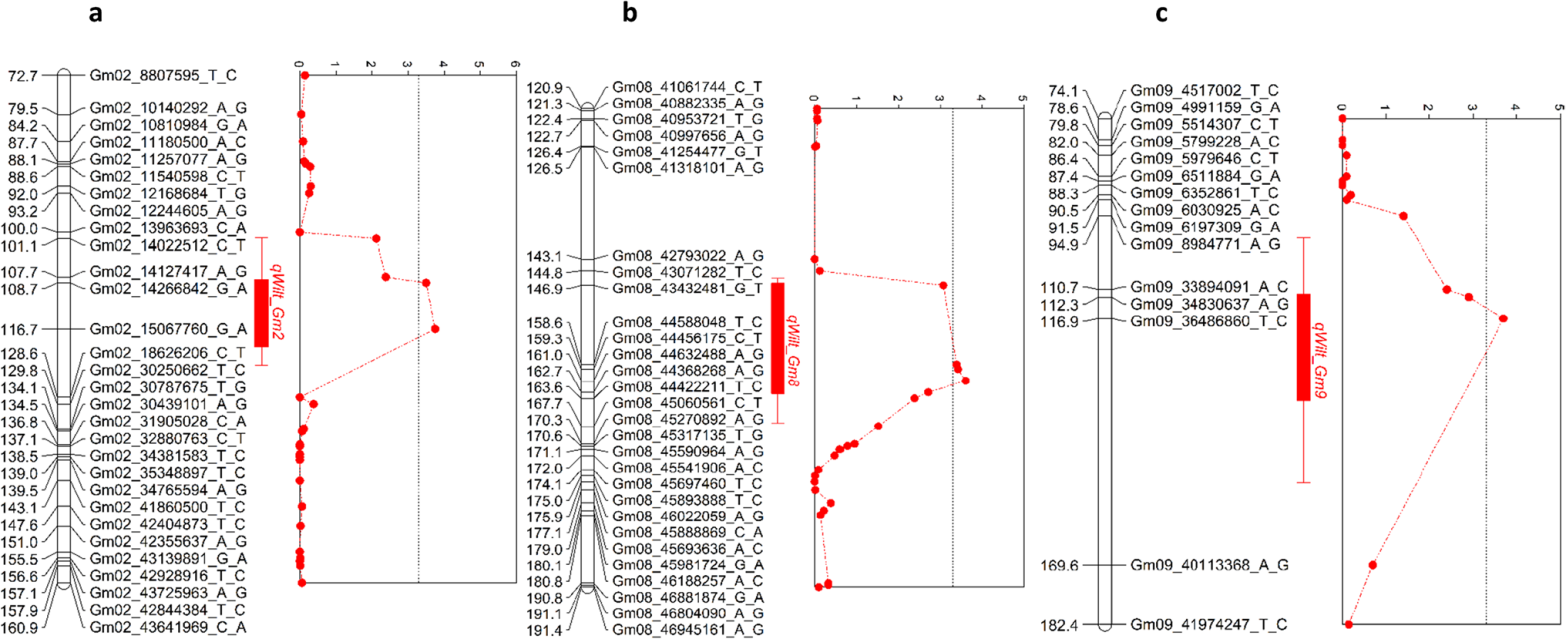
Composite interval mapping for canopy wilting in the recombinant inbred line population derived from Hutcheson × PI 471938 in the combined environments. Genetic maps with cM positions for chromosomes with QTLs meeting logarithm of odds (LOD) significance thresholds of 3.3 which is indicated by the dotted black lines. **A** Segment of chromosome 2 harboring the *qWilt_Gm2*; **B** Segment of chromosome 8 harboring the *qWilt_Gm8*; **C** Segment of chromosome 9 harboring the* qWilt_Gm9*

In the combined analysis across environments, three QTLs were identified on Chrs 2, 8, and 9. These QTLs accounted for between 10 and 14% of the phenotypic variation observed for slow canopy wilting. On Chr 2, the QTL, *qWilt_Gm2*, was identified and explained 11% of the phenotypic variation observed in canopy wilting (Table 2). The peak marker for *qWilt_Gm2* was Gm02_15067760_G_A, which was located at 15,271,225 bp and the confidence interval (CI) of this QTL spanned 4.7 Mb (14,220,378–18,913,725 bp) (Fig. 2a). A QTL was identified on Chr 8 in the combined environments (*qWilt_Gm8*) and accounted for 10% of the phenotypic variation observed in slow canopy wilting (Table 2). The QTL *qWilt_Gm8* spanned a CI of 1.5 Mb (44,267,551–45,913,059 bp) on Chr 8 (Fig. 2b, Table 2). The peak marker for *qWilt_Gm8* was Gm08_44368268_A_G (45,403,652 bp). The QTL on Chr 9 *(qWilt_Gm9*) was identified in the combined analysis, which explained 14% of the observed variance in the wilting score (Table 2). The peak of *qWilt_Gm9* was at marker Gm09_36486860_T_C (39,047,264 bp) with a CI of 6.3 Mb (36,455,035–42,790,738 bp) (Fig. 2c, Table 2). From the combined analysis, QTLs on Chrs 2, 8, and 9 all had a positive allelic effect (Table 2). Positive additive effects indicate that the mean canopy wilting score for the RILs possessing the allele from PI 471938 was lower than those possessing the alleles from Hutcheson.

**Table 2:** QTLs for canopy wilting that were identified by composite interval mapping (CIM) for the Hutcheson × PI 471938 RIL population in the combined environments

QTL name	Chr^a^	Peak marker	Pos (cM)^b^	CI (cM)^c^	Pos (bp)^d^	CI (bp)^e^	LOD^f^	Additive Effect^g^	R^2^	Source of favorable Allele
*qWilt_Gm2*	2	Gm02_15067760_G_A	114.8	102.5–127.7	15,271,225	14,220,378–18913725	3.9	1.00	0.11	PI 471938
*qWilt_Gm8*	8	Gm08_44368268_A_G	162	146.9–167.9	45,403,652	44,267,551–45,913,059	3.6	0.95	0.10	PI 471938
*qWilt_Gm9*	9	Gm09_36486860_T_C	116.9	105.6–162.7	39,047,264	36,455,035–42790738	3.7	0.98	0.14	PI 471938

^a^Chromosome

^b^Position in centiMorgans based on the genetic map

^c^Confidence interval in centiMorgans which includes all SNPs that met logarithm of the odds (LOD) threshold

^d^Glyma.Wm82.a2 physical position of the peak SNP marker

^e^Confidence interval based on Glyma.Wm82.a2 physical positions of all SNPs that met logarithm of the odds (LOD) threshold

^f^Logarithm of the odds (LOD) of peak SNP marker. The significance LOD threshold (LOD = 3.3) was determined by 1000 permutations, with a significance level of α = 0.05

^g^Additive allelic effect

## Discussion

Slow canopy wilting is one of indicators for drought tolerance, which could lead to less yield reduction during drought stress (Sloane et al. 1990). It has been extensively used for phenotyping for gene discovery and breeding selection (Abdel-Haleem et al. 2012; Carter et al. 2016; Kaler et al. 2017; and Steketee et al. 2020). Additionally, traits such as root architecture and canopy temperature have also been evaluated for drought stress tolerance (Tuberosa 2012; Mace et al. 2012; Fenta et al. 2014; Zhou et al. 2020). Because it possesses a slow canopy wilting trait, PI 471938 has been used extensively in the southern breeding programs and is in the pedigree of over 30 lines that have been evaluated for potential commercial use. These lines were advanced to USDA Uniform Soybean Tests, indicating that they possessed potential as commercial cultivars for use in the Southern USA. An example is USDA-N8002, which was derived from both PI 471938 (25% by pedigree) and PI 416937 (12.5% by pedigree) and possesses drought-tolerant traits with high yield potential (Carter et al. 2016). This cultivar has become a valuable source for the slow wilting trait, which demonstrates that breeding for drought tolerance can succeed by incorporation of the slow canopy wilting trait from plant introductions.

Slow canopy wilting in soybean is potentially related to many possible physiological mechanisms, which are complex. This study identified three QTLs on Chrs 2, 8, and 9 using a combined analysis that each explains a relatively small portion of phenotypic variation (10–14%) that was observed in canopy wilting. In addition, six QTLs were identified only in a single environment. This indicated that the genetic architecture of the slow canopy wilting in PI 471938 could be governed by many minor QTLs which are not stable across environments.

Although the genotype by environment interactions were significant (*p* < 0.05) (Supplementary Table S2), the severity of wilting experienced in the seven environments varied (Fig. 1). The correlations (*r* = 0.22 to 0.38) of wilting scores between locations in a given year were significant (*p* < 0.05), except for the 2016_GA and 2016_KS locations (*r* = 0.07), indicating that the genotypes tested wilted similarly across the locations for a given year (Table 1). However, four pairs of environments across years showed insignificant negative correlations in wilting scores. Using CIM, eight QTLs were identified for canopy wilting in six of the seven individual environments tested (Supplementary Table S3). No significant QTL was detected in the 2018_NC environment at a LOD = 3.4. These QTLs accounted for 8 to 20% of the phenotypic variation that was observed in an individual environment (Supplementary Table S3). Heritability (0.29) for the canopy wilting trait on the entry-mean basis across environments was relatively low, suggesting the complexity of the canopy wilting trait. However, it was comparable to those observed in previously canopy wilting QTL mapping and GWAS studies (Charlson et al. 2009; Abdel-Haleem et al. 2012; Hwang et al. 2015; Kaler et al. 2017).

The QTLs *qWilt_Gm2* and *qWilt_Gm8* identified in the combined analysis were located in similar regions as QTLs identified in the individual environments (Table 2 and Supplementary Table S3). The remaining six QTLs were identified in only one environment. This is most likely due to the highly complex nature of the slow canopy wilting and the environmental variance that affected these QTLs. The QTL *qWilt_Gm2* is located approximately 1.9 Mb upstream of the peak of the meta-QTL *mqCanopywilt-003* identified by Hwang et al. (2016). It is also located in a similar genomic region to the significant QTL *qSW-Gm02* that was identified by Abdel-Haleem et al. (2012) in a RIL population derived from Benning × PI 416937. The peak of *qWilt_Gm8* on Chr 8 was located 199 kb from the significant SNP Gm08_44751317_C_T identified in a GWAS for canopy wilting (Kaler et al. 2017). The Chr 9 QTL, *qWilt_Gm9,* was located 2.3 Mb from a reported significant SNP identified in the GWAS for canopy wilting done by Kaler et al. (2017) and 2.1 Mb from the GWAS performed by Steketee et al. (2020). The QTLs identified in this study are in similar genomic regions reported in previous mapping and association studies (Abdel-Haleem et al. 2012; Hwang et al. 2016; Kaler et al. 2017). However, these QTLs only provide a small portion of the genetic control of the slow canopy wilting trait from PI 471938 and this is most likely due to the highly complex nature of the slow canopy wilting trait. It is noted that the size of RIL population is relatively small, which may affect the detection of some minor QTLs.

Genomic regions significantly associated with slow canopy wilting QTLs were searched for candidate genes within the QTL confidence intervals (Table 2) based on the peak markers, Gm02_15067760_G_A, Gm08_44368268_A_G and Gm09_36486860_T_C, respectively. A total of 235 candidate genes were found in the *qWilt_Gm2* region on Chr 2. Of these genes, 16 candidate genes were reported in the literature, which were related to the drought tolerance (Supplementary Table S4). This included candidate genes for calmodulin-like 11, protein phosphatase 2C family protein, lipid transfer protein, zinc finger protein, and aluminum-activated malate transporters that plays a role in cell signaling of abiotic stress (Shelp et al. 2012; Yang et al. 2012; Shinozaki et al. 2007, Scholz et al. 2015; Ramesh et al. 2018; Zhang et al. 2021). For the QTL *qWilt_Gm8* on Chr 8, a total of 193 genes were in the confidence interval and 34 candidate genes were found to be related to drought tolerance (Supplementary Table S4) based on the published information (Shinozaki et al. 2007; Shao et al. 2008; Cho et al. 2009; Guan et al. 2013; Luo et al. 2019; Ao et al. 2022). They included one chaperone Dnaj-domain superfamily protein gene, two RNA-binding KH domain-containing protein genes, six clustered UDP-glycosyltransferase superfamily protein genes, and one calcium ion transport, transmembrane transport, and protein binding gene. *Glyma.08g337000* encodes Ca^2+^ exchange proteins in Arabidopsis, which helps regulate stomatal movements (Cho et al. 2009). The Glyma.*08g337100* gene encodes a chaperone DnaJ-domain superfamily protein. In rice grown under drought stress, proteins in this family showed elevated transcription under drought stress (Luo et al. 2019). Fifty-six candidate genes were reported in the literature related to the drought tolerance from a list of 567 candidate genes found in the interval of *qWilt_Gm9* QTL (Supplementary Table S4), including nucleotide-diphospho-sugar transferase family protein, sucrose synthase 6, and NAC domain-containing protein 57 (González et al. 1995; Shinozaki et al. 2007; and Zhang et al. 2021). The candidate gene, *Glyma.09g166400*, encodes an organic cation/carnitine transporter. In Arabidopsis, organic cation/carnitine transporters were involved in lateral root formation, and plants containing knockouts of this gene exhibited increased root growth (Lelandais-Brière et al. 2007). The candidate genes that were identified around each of three QTLs that were present in the combined analysis are related to stress tolerance in soybean or other crops. These candidate genes could provide targets for further studies to identify the mechanism that underlies drought tolerance in PI 471938.

Breeding for drought tolerance is a complex process. It requires adequate, repeatable drought stress every year to effectively evaluate large numbers of genotypes in field environments. As shown in this experiment, relying on natural drought can be difficult. In two out of four years during this study, at least one location did not experience any drought stress. Lack of consistent phenotyping, combined with the labor and resources needed to evaluate large numbers of soybean breeding lines, makes conducting drought field experiments challenging. The results from this study could allow for the use of QTLs and marker information to aid in the selection of lines for the slow canopy wilting trait in lieu of field phenotyping experiments. Based on this and previous research, it is obvious that drought tolerance is a highly quantitative trait with many underlying mechanisms that lead to the slow canopy wilting phenotype. Slow canopy wilting only is one of the indicators for screening drought tolerance in the field (Ye et al. 2020). Selection of QTLs with small effects that are not consistent across environments could prove difficult. Genomic selection has become a useful tool to select quantitative traits in breeding programs (Miller et al. 2023a and 2023b). These QTLs from PI 471938 for slow canopy wilting could be incorporated into the genomic selection models to help predict the performance of lines derived from PI 471938 for drought tolerance without having to phenotype them under drought stress in the early generations. This would relieve the need to have consistent environmental conditions to effectively phenotype breeding populations and would allow for the deployment of these QTLs in new cultivars more efficiently where the slow wilting trait could help soybean producers limit the significant effects of drought in soybean production.

## Conclusions

Using an RIL population derived from Hutcheson × PI 471938, three QTLs on Chrs 2, 8, and 9 for slow canopy wilting were identified through a combined analysis across environments with each accounting for 10—14% of the phenotypic variation in wilting response. Six QTLs were identified in only one environment. This showed that the complex nature of the slow canopy wilting and the environmental effects on the trait. The genomic locations of these three QTLs identified in this study are in proximity to those previously reported in the mapping and GWAS results. The candidate genes located near all three QTLs are targets for further studies to understand the functions of these genes that control slow canopy wilting in PI 471938. The QTLs discovered in this study will allow for improved efficiency in breeding drought-tolerant soybeans, through marker-assisted selection or genomic selection. These improved drought-tolerant cultivars can then be used by soybean producers to meet the climate challenges that they face due to drought.

### Supplementary Information

Below is the link to the electronic supplementary material.

Supplementary file1 (DOCX 16 KB)

Supplementary file2 (XLSX 17 KB)

## Data Availability

The dataset generated from this study is available from the corresponding author upon request.

## References

[CR1] Abdel-Haleem H, Carter TE, Purcell LC (2012). Mapping of quantitative trait loci for canopy-wilting trait in soybean [*Glycine max* (L). Merr]. Theor Appl Genet.

[CR2] Ao B, Han Y, Wang S, Wu F, Zhang J (2022). Genome-wide analysis and profile of UDP-glycosyltransferases family in alfalfa (Medicago sativa L) under drought stress. Int J Mol Sci.

[CR3] Bagherzadi L, Sinclair TR, Zwieniecki M (2017). Assessing water-related plant traits to explain slow-wilting in soybean PI 471938. J Crop Improv.

[CR4] Brim CA (1966). A modified pedigree method of selection in soybeans. Crop Sci.

[CR5] Buss GR, Camper HM, Roane CW (1988). Registration of “Hutcheson” soybean. Crop Sci.

[CR6] Carter TE, Todd SM, Gillen AM (2016). Registration of ‘USDA-N8002’ soybean cultivar with high yield and abiotic stress resistance traits. Plant Regist.

[CR7] Carter TE, de Souza PI, Purcell LC (1999) Recent advances in breeding for drought and aluminum resistance in soybean. In: Kauffman H (ed) World soybean conference VI. Champaign, IL, pp 106–125

[CR8] Chamarthi SK, Kaler AS, Abdel-Haleem H, Fritschi FB, Gillman JD, Ray JD, Smith JR, Dhanapal AP, King CA, Purcell LC (2021). Identification and confirmation of loci associated with canopy wilting in soybean using genome-wide association mapping. Front Plant Sci.

[CR9] Charlson D, Bhatnagar S, King CA (2009). Polygenic inheritance of canopy wilting in soybean [*Glycine max* (L.) Merr.]. Theor Appl Genet.

[CR10] Cho D, Kim SA, Murata Y (2009). De-regulated expression of the plant glutamate receptor homolog AtGLR3.1 impairs long-term Ca2+-programmed stomatal closure. Plant J.

[CR11] Devi MJ, Sinclair TR (2013). Nitrogen fixation drought tolerance of the slow-wilting soybean PI 471938. Crop Sci.

[CR12] Devi JM, Sinclair TR, Chen P, Carter TE (2014). Evaluation of elite southern maturity soybean breeding lines for drought-tolerant traits. Agron J.

[CR13] Fenta BA, Beebe SE, Kunert KJ, Burridge JD, Barlow KM, Lynch JP, Foyer CH (2014). Field phenotyping of soybean roots for drought stress tolerance. Agronomy.

[CR14] Fletcher AL, Sinclair TR, Allen LH (2007). Transpiration responses to vapor pressure deficit in well-watered ‘slow-wilting’ and commercial soybean. Environ Exp Bot.

[CR15] González EM, Gordon AJ, James CL, Arrese-Lgor C (1995). The role of sucrose synthase in the response of soybean nodules to drought. J Exp Bot.

[CR16] Guan Q, Wen C, Zeng H, Zhu J (2013). A KH domain-containing putative RNA-binding protein is critical for heat stress-responsive gene regulation and thermotolerance in Arabidopsis. Mol Plant.

[CR17] Guenther JF, Chanmanivone N, Galetovic MP, Wallace IS, Cobb JA, Roberts DM (2003). Phosphorylation of soybean nodulin 26 on serine 262 enhances water permeability and is regulated developmentally and by osmotic signals. Plant Cell.

[CR18] Holland JB, Nyquist WE, Cervantes-Martínez CT, Janick J (2003). Estimating and interpreting heritability for plant breeding: an update. Plant breeding reviews.

[CR19] Hudak CM, Patterson RP (1996). Root distribution and soil moisture depletion pattern of a drought-resistant soybean plant introduction. Agron J.

[CR20] Hufstetler EV, Boerma HR, Carter TE, Earl HJ (2007). Genotypic variation for three physiological traits affecting drought tolerance in soybean. Crop Sci.

[CR21] Hwang S, King CA, Ray JD (2015). Confirmation of delayed canopy wilting QTLs from multiple soybean mapping populations. Theor Appl Genet.

[CR22] Hwang S, King CA, Chen P (2016). Meta-analysis to refine map position and reduce confidence intervals for delayed-canopy-wilting QTLs in soybean. Mol Breed.

[CR23] Institute SAS (2014). The SAS system for Windows. Release.

[CR24] Kaler AS, Ray JD, Schapaugh WT (2017). Genome-wide association mapping of canopy wilting in diverse soybean genotypes. Theor Appl Genet.

[CR25] Khan MN, Komatsu S (2016). Proteomic analysis of soybean root including hypocotyl during recovery from drought stress. J Proteomics.

[CR26] Lelandais-Brière C, Jovanovic M, Torres GAM (2007). Disruption of AtOCT1, an organic cation transporter gene, affects root development and carnitine-related responses in Arabidopsis. Plant J.

[CR27] Liu XS, Liang CC, Hou SG, Wang X, Chen DH, Shen JL, Zhang W, Wang M (2020). The LRR-RLK protein HSL3 regulates stomatal closure and the drought stress response by modulating hydrogen peroxide homeostasis. Front Plant Sci.

[CR28] Lu G, Wang X, Liu J, Yu K, Gao Y, Liu H, Wang C, Wang W, Wang G, Liu M, Mao G (2014). Application of T-DNA activation tagging to identify glutamate receptor-like genes that enhance drought tolerance in plants. Plant Cell Rep.

[CR29] Lu P, Magwanga RO, Kirungu JN, Hu Y, Dong Q, Cai X, Zhou Z, Wang X, Zhang Z, Hou Y, Wang K (2019). Overexpression of cotton a DTX/MATE gene enhances drought, salt, and cold stress tolerance in transgenic Arabidopsis. Front Plant Sci.

[CR30] Luo Y, Fang B, Wang W (2019). Genome-wide analysis of the rice J-protein family: identification, genomic organization, and expression profiles under multiple stresses. 3 Biotech.

[CR31] Mace ES, Singh V, van Oosterom EJ (2012). QTL for nodal root angle in sorghum (Sorghum bicolor L. Moench) co-locate with QTL for traits associated with drought adaptation. Theor Appl Genet.

[CR32] Miller MJ, Song Q, Fallen B, Li Z (2023). Genomic prediction of optimal cross combinations to accelerate genetic improvement of soybean (Glycine max). Front Plant Sci.

[CR33] Miller MJ, Song Q, and Li Z (2023b) Genomic selection of soybean (Glycine max) for genetic improvement of yield and seed composition in a breeding context. Plant Genome p.e20384. 10.1002/tpg2.20384 PMC12807242 37749946

[CR34] Pantalone V, Rebetzke G (1996). Phenotypic evaluation of root traits in soybean and applicability to plant breeding. Crop Sci.

[CR35] Pathan SM, Lee J-D, Sleper DA (2014). Two soybean plant introductions display slow leaf wilting and reduced yield loss under drought. J Agron Crop Sci.

[CR36] Purcell LC, Specht JE (2004) Physiological traits for ameliorating drought stress. In: Soybeans: improvement, production, and uses. pp 569–620

[CR37] Ramesh SA, Kamran M, Sullivan W (2018). Aluminum-activated malate transporters can facilitate GABA transport. Plant Cell.

[CR38] Rao MJ, Xu Y, Tang X, Huang Y, Liu J, Deng X, Xu Q (2020). CsCYT75B1, a Citrus CYTOCHROME P450 gene, is involved in accumulation of antioxidant flavonoids and induces drought tolerance in transgenic Arabidopsis. Antioxidants.

[CR39] Riar MK, Cerezini P, Manandhar A (2018). Expression of drought-tolerant N fixation in heterogeneous inbred families derived from PI471938 and Hutcheson soybean. Crop Sci.

[CR40] Ristic Z, Yang G, Martin B, Fullerton S (1998). Evidence of association between specific heat-shock protein (s) and the drought and heat tolerance phenotype in maize. J Plant Phys.

[CR41] Rivers RL, Dean RM, Chandy G, Hall JE, Roberts DM, Zeidel ML (1997). Functional analysis of nodulin 26, an aquaporin in soybean root nodule symbiosomes. J Biol Chem.

[CR42] Sadok W, Gilbert ME, Raza MAS, Sinclair TR (2012). Basis of slow-wilting phenotype in soybean PI 471938. Crop Sci.

[CR43] Scholz SS, Reichelt M, Vadassery J, Mithöfer A (2015). Calmodulin-like protein CML37 is a positive regulator of ABA during drought stress in Arabidopsis. Plant Signal Behav.

[CR44] Shao HB, Song WY, Chu LY (2008). Advances of calcium signals involved in plant anti-drought. CR Biol.

[CR45] Shelp BJ, Bozzo GG, Zarei A (2012). Strategies and tools for studying the metabolism and function of γ-aminobutyrate in plants. II. Integrated analysis. Botany.

[CR46] Shinozaki K, Yamaguchi-Shinozaki K (2007). Gene networks involved in drought stress response and tolerance. J Exp Bot.

[CR47] Sinclair TR, Purcell LC, Vadez V (2000). Identification of soybean genotypes with N fixation tolerance to water deficits. Crop Sci.

[CR48] Sloane RJ, Patterson RP, Carter TE (1990). Field drought tolerance of a soybean plant introduction. Crop Sci.

[CR49] Song Q, Jenkins J, Jia G, Hyten DL, Pantalone V, Jackson SA, Schmutz J, Cregan PB (2016). Construction of high-resolution genetic linkage maps to improve the soybean genome sequence assembly Glyma1. 01. BMC Genomics.

[CR50] Song Q, Yan L, Quigley C, Fickus E, Wei H, Chen L, Dong F, Araya S, Liu J, Hyten D, Pantalone V (2020). Soybean BARCSoySNP6K: an assay for soybean genetics and breeding research. Plant J.

[CR51] Soystats (2020) A reference guide to important soybean facts & figures. http://soystats.com

[CR52] Specht J, Hume D, Kumudini S (1999). Soybean yield potential—A genetic and physiological perspective. Crop Sci.

[CR53] Specht JE, Diers BW, Nelson RL, et al (2015) Soybean. In: Yield gains in major U.S. Field Crops. pp 311–355. Wiley-Blackwell

[CR54] Steketee CJ, Schapaugh WT, Carter TE, Li Z (2020). Genome-wide association analyses reveal genomic regions controlling canopy wilting in soybean. G3: Genes Genomes, Genet.

[CR55] Tanaka Y, Fujii K, Shiraiwa T (2010). Variability of leaf morphology and stomatal conductance in soybean [*Glycine* max (L.) Merr.] cultivars. Crop Sci.

[CR56] Tuberosa R (2012). Phenotyping for drought tolerance of crops in the genomics era. Front Physiol.

[CR57] van Ooijen JW (2006). JoinMap® 4: Software for the calculation of genetic linkage maps in experimental populations.

[CR58] Voorrips RE (2002). MapChart: Software for the graphical presentation of linkage maps and QTLs. J Hered.

[CR59] Wang S, Basten CJ and Zeng CB (2012) Windows QTL cartographer 2.5. Department of Statistics, North Carolina State University, Raleigh, NC. (http://statgen.ncsu.edu/qtlcart/WQTLCart.htm)

[CR60] Yang ZB, Eticha D, Albacete A, Rao IM, Roitsch T, Horst WJ (2012). Physiological and molecular analysis of the interaction between aluminium toxicity and drought stress in common bean (*Phaseolus vulgaris*). J Exp Bot.

[CR61] Ye H, Song L, Schapaugh WT, Ali ML, Sinclair TR, Riar MK, Mutava RN, Li Y, Vuong T, Valliyodan B, Pizolato Neto A (2020). The importance of slow canopy wilting in drought tolerance in soybean. J Exp Bot.

[CR62] Zhang Q, Li J, Zhang W, Yan S, Wang R, Zhao J, Li Y, Qi Z, Sun Z, Zhu Z (2012). The putative auxin efflux carrier OsPIN3t is involved in the drought stress response and drought tolerance. Plant J.

[CR63] Zhang J, Huang D, Zhao X, Zhang M (2021). Evaluation of drought resistance and transcriptome analysis for the identification of drought-responsive genes in Iris germanica. Sci Rep.

[CR64] Zhou J, Zhou J, Ye H, Ali ML, Nguyen HT, Chen P (2020). Classification of soybean leaf wilting due to drought stress using UAV-based imagery. Comput Electron Agric.

