# Supplementary material for: Genetic mapping reveals the complex genetic architecture controlling slow canopy wilting in soybean
Source: Theor Appl Genet. 2024 Apr 17;137(5):107. doi: 10.1007/s00122-024-04609-w (PMC11024021; doi:10.1007/s00122-024-04609-w)
Supplement: Supplementary file 1 — Supplementary file1 (DOCX 16 KB) [file 122_2024_4609_MOESM1_ESM.docx]

**Supplemental Tables**

Supplemental Table S1. Summary of phenotyping environments for the Hutcheson × PI 471938 recombinant inbred line (RIL) population.

| Environment | Location | No. of rows per plot | No. of reps | Length of plots (m) | Number of raters |
| --- | --- | --- | --- | --- | --- |
| 2016_GA | Athens, GA | 2 | 3 | 1.8 | Mean of three ratings |
| 2016_KS | Salina, KS | 2 | 2 | 3.6 | Single rating |
| 2018_GA | Midville, GA | 2 | 3 | 1.8 | Single rating |
| 2018_KS | Salina, KS | 2 | 3 | 3.6 | Single rating |
| 2018_NC | Sandhills, NC | 2 | 3 | 1.8 | No rating |
| 2019_GA | Midville, GA | 2 | 3 | 1.8 | Single rating |
| 2019_KS | Salina, KS | 2 | 3 | 3.6 | Single rating |
| 2019_NC | Sandhills, NC | 2 | 3 | 1.8 | Single rating |

Supplemental Table S2. Analysis of variance (ANOVA) for effects of genotype (G), environment (E), and their interaction based on the RIL population canopy wilting scores. The G × E mean square was used as the denominator of the F Value for significance testing.

| Source | DF | F Value | P-value (> F) |
| --- | --- | --- | --- |
| Genotype (G) | 129 | 2.38 | <0.0001 |
| Environment (E) | 6 | 32.5 | <0.0001 |
| G × E | 774 | 1.68 | <0.0001 |

Supplemental Table S3. QTLs for canopy wilting that were identified with composite interval mapping (CIM) for the Hutcheson × PI 471938 RIL population in the individual environments.

| QTL Name | Chr^a^ | Peak Marker | Pos (cM)^b^ | CI (cM)^c^ | Pos (bp)^d^ | CI (bp)^e^ | LOD^f^ | Effect^g^ | R^2^ | Env^h^ |
| --- | --- | --- | --- | --- | --- | --- | --- | --- | --- | --- |
| *qWilt_Gm2.1* | 2 | Gm02_12,244,605_A_G | 97.2 | 88.3-107.3 | 12582331 | 11349126-14470563 | 5.1 | 1.6 | 0.13 | Salina, KS |
| *qWilt_Gm7* | 7 | Gm07_6,781,309_T_C | 45.3 | 43.3-50.9 | 6819959 | 6898693- 7409086 | 4.2 | 1.7 | 0.11 | Sandhill, NC |
| *qWilt_Gm8* | 8 | Gm08_44,632,488_A_G | 160.3 | 146.9-169.7 | 45140042 | 44267551-45913059 | 3.6 | 1.8 | 0.10 | Salina, KS |
| *qWilt_Gm13_1.1* | 13 | Gm13_29,481,243_C_A | 125.2 | 123.7-127.2 | 30681387 | 30117998-30875555 | 6.0 | -1.7 | 0.13 | Athens, GA |
| *qWilt_Gm13_1.2* | 13 | Gm13_39,978,113_C_A | 244.2 | 230.5-251.4 | 41157215 | 39311323-42650588 | 4.0 | -1.4 | 0.13 | Athens, GA |
| *qWilt_Gm13.3* | 13 | Gm13_38,133,840_A_C | 229.9 | 222.6-238.8 | 39311323 | 38159424- 39797053 | 7.9 | 2.1 | 0.20 | Midville, GA |
| *qWilt_Gm15* | 15 | Gm15_6,925,513_T_C | 39.7 | 38.2-42.3 | 6939367 | 6841404- 7358153 | 3.8 | -1.4 | 0.08 | Midville, GA |
| *qWilt_Gm16* | 16 | Gm16_7,851,145_G_A | 58.9 | 51.9-63.4 | 8008387 | 6858504- 8197236 | 4.5 | -1.0 | 0.11 | Salina, KS |

^a^ Chromosome

^b^ Position in centiMorgans based on the genetic map

^c^ Confidence interval in centiMorgans which includes all SNPs that met logarithm of the odds (LOD) threshold

^d^ Glyma.Wm82.a2 physical position of peak SNP marker

^e^ Confidence interval based on Glyma.Wm82.a2 physical positions of all SNPs that met logarithm of the odds (LOD) threshold

^f^ Logarithm of the odds (LOD) of peak SNP marker

^g^ Additive allelic effect

^h^ Environment
